# Interaction of healthcare staff’s attitude with barriers to physical activity in hemodialysis patients: A quantitative assessment

**DOI:** 10.1371/journal.pone.0196313

**Published:** 2018-04-27

**Authors:** Giuseppe Regolisti, Umberto Maggiore, Alice Sabatino, Ilaria Gandolfini, Sarah Pioli, Claudia Torino, Filippo Aucella, Adamasco Cupisti, Valentina Pistolesi, Alessandro Capitanini, Giorgia Caloro, Mariacristina Gregorini, Yuri Battaglia, Marcora Mandreoli, Lucia Dani, Giovanni Mosconi, Vincenzo Bellizzi, Biagio Raffaele Di Iorio, Paolo Conti, Enrico Fiaccadori, on behalf of the Gruppo di Studio “Esercizio fisico nel paziente con insufficienza renale cronica” of the Società Italiana di Nefrologia

**Affiliations:** 1 Unità di Fisiopatologia dell’Insufficienza Renale, and Scuola di Specializzazione in Nefrologia, Università di Parma, Parma, Italy; 2 Centro Trapianti Rene-Pancreas, Azienda Ospedaliera-Universitaria di Parma, Parma, Italy; 3 Nefrologia ASL Parma, Parma, Italy; 4 Nefrologia Dialisi e Trapianto, Reggio Calabria, Italy; 5 Nefrologia e Dialisi, IRCCS Casa Sollievo della Sofferenza, San Giovanni Rotondo, Foggia, Italy; 6 Dipartimento di Medicina Clinica e Sperimentale, Università di Pisa, Pisa, Italy; 7 SSD Dialisi, Policlinico, Roma, Italy; 8 Nefrologia, Pistoia, Italy; 9 Nefrologia Dialisi e Trapianto, Bari, Italy; 10 Nefrologia e Dialisi ASMN, Reggio Emilia, Italy; 11 Nefrologia, Ferrara, Italy; 12 Nefrologia e Dialisi, Imola, Italy; 13 Nefrologia e Dialisi, San Miniato, Empoli, Italy; 14 Nefrologia e Dialisi, Forlì, Italy; 15 Nefrologia e Dialisi, Salerno, Italy; 16 Nefrologia e Dialisi AOSG Moscati, Avellino, Italy; 17 Azienda USL Toscana Sud Est, Nefrologia e Dialisi, Grosseto, Italy; Hospital Universitario de la Princesa, SPAIN

## Abstract

**Background and aim of the study:**

In hemodialysis patients, sedentarism is a potentially modifiable mortality risk factor. We explored whether healthcare staff’s attitude towards exercise interacts with patient-perceived barriers in modifying the level of physical activity in this population.

**Methods:**

In this prospective, cross-sectional, multicenter study we recruited 608 adult patients and 330 members of the healthcare staff in 16 hemodialysis units in Italy. We assessed patient-perceived barriers to, and healthcare staff’s attitude towards, exercise by specific questionnaires. We fitted multilevel linear models to analyze the relationships of either barriers or staff’s attitude, and their interaction, with a measure of patient self-reported physical activity (the Human Activity Profile–Adjusted Activity Score [HAP-AAS]), adjusting for multiple confounders. We also employed latent class analysis to dichotomize patients into those endorsing or not endorsing barriers.

**Results:**

Most barriers were negatively associated with the HAP-AAS (adjusted change attributable to a given barrier ranging between -5.1 [“Feeling too old”, 95% Confidence Interval: -9.4 to -0.8] and -15.6 [“Ulcers on legs and feet”, 95%CI: -24.8 to -6.5]. We found a significant interaction between staff’s attitude and barriers (adjusted P values ranging between 0.03 [“I do not believe that it is physician’s or nurse’s role providing advice on exercise to patients on dialysis”] and 0.001 [“I do not often ask patients about exercise”]). A beneficial effect of a proactive staff’s attitude was evident only in patients not endorsing barriers.

**Conclusions:**

Barriers and non-proactive staff’s attitude reduce physical activity in hemodialysis patients. Patients not endorsing barriers benefit the most from a proactive staff’s attitude.

## Introduction

In patients with end-stage kidney disease on hemodialysis the degree of physical activity is low, even if compared to age-matched sedentary subjects [1]. Sedentarism is an important factor of frailty and disability in patients with chronic kidney disease (CKD) [2], especially in those on routine hemodialysis [3]. Thus, it is not surprising that impaired mobility and reduced physical activity emerged as powerful predictors of increased cardiovascular risk and mortality in patients with CKD, and particularly in those on hemodialysis [4–7].

In recent years, there has been a growing interest towards barriers hindering physical activity in hemodialysis patients [8–13]; these barriers include both disease-specific (i.e., comorbidities) and patient-specific (i.e., physical, psychological, cultural, and socio-economic) conditions. Furthermore, the specific attitude and beliefs of members of the healthcare staff concerning exercise in hemodialysis patients could significantly contribute to physical inactivity in this population [13–16]. For example, counseling on the implementation of exercise is low among nephrologists [17], while a proactive approach by specialized teams may help in the empowerment and motivation of patients [18]. To date, the assessment of the effects of healthcare staff’s attitude on patients’ level of physical activity has been attempted only in qualitative studies using purposive sampling and thematic saturation [13], focus groups and semi-structured interviews [15,19,20]. However, it is not known whether a proactive staff’s attitude may directly and quantitatively modify the effects of patient-perceived barriers on the level of physical activity in this population.

Thus, we planned a prospective, cross-sectional, multicenter study aimed at a quantitative evaluation of the effects of the interaction between healthcare staff’s attitude towards exercise and patient-perceived barriers on the level of self-reported degree of physical activity in hemodialysis patients. We hypothesized that a proactive staff’s attitude may attenuate the negative effect of barriers.

## Material and methods

### Patients

This study was planned and conducted as a spontaneous observational study under the auspices of the “Physical Exercise in Patients with CKD” Study Group of the Italian Society of Nephrology. From January 1^st^ 2013 to December 31^st^ 2015 we screened 1379 patients on routine hemodialysis at 16 dialysis units in 6 regions covering most of the Italian peninsular territory; all of the participating dialysis units were public institutions belonging to the Italian National Healthcare Delivery System. Among these centers, 14 were hospital-based and 2 were satellite units. Five centers had specific expertise on the implementation of structured exercise programmes in dialysis patients, and had previously participated in a large interventional study on the effects of exercise training on physical performance in hemodialysis patients [21]. Written informed consent was obtained from all participants. Previous stroke with neurological sequelae, amputation, and inability to provide informed consent represented exclusion criteria.

**Fig 1:**
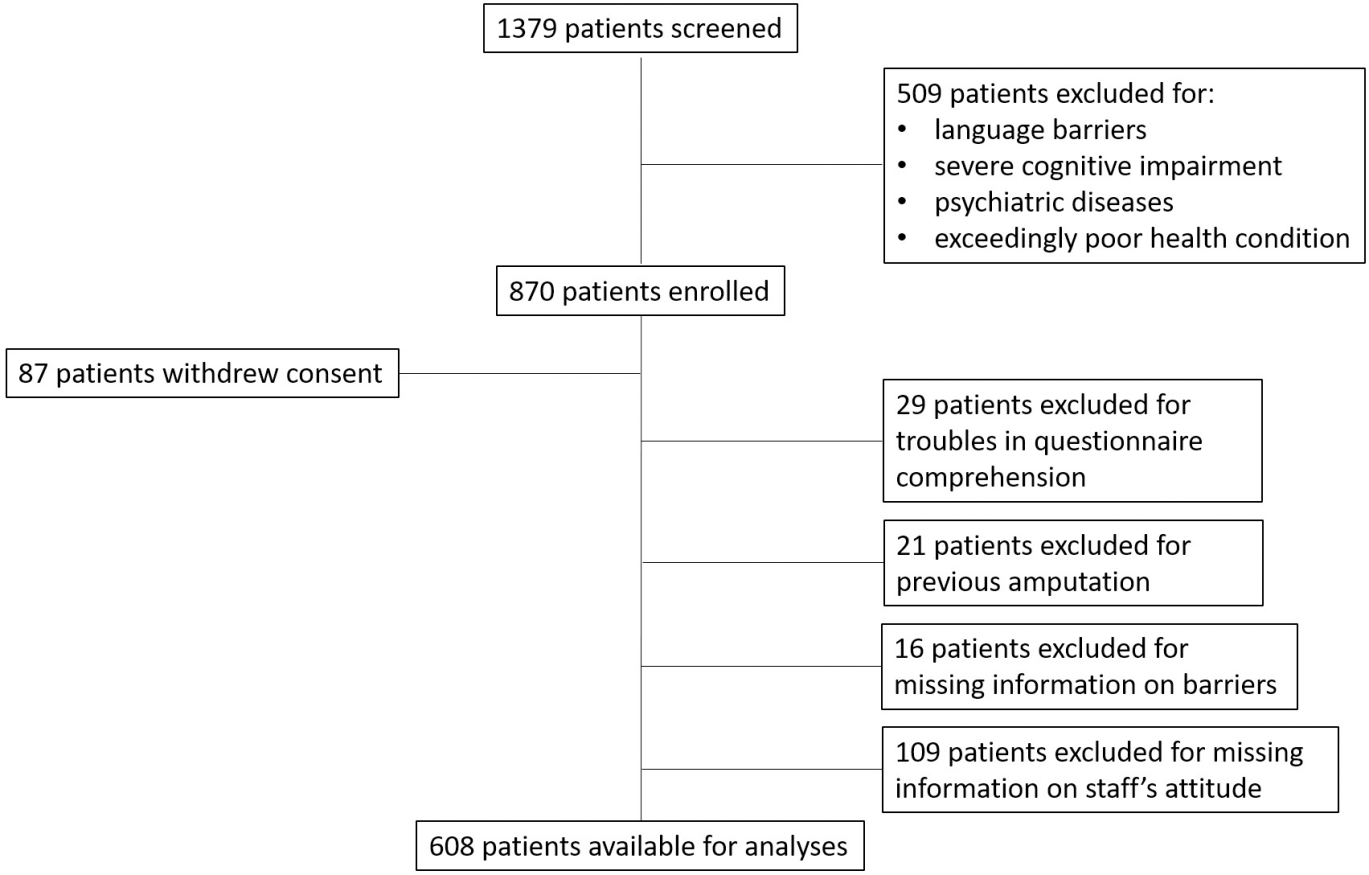
Process of patient selection.

Of the 1379 patients originally screened, 509 were initially excluded because of language barriers, severe cognitive impairment or psychiatric diseases, or exceedingly poor health condition; thus, 870 patients were enrolled. Eighty-seven patients subsequently withdrew consent, 29 had troubles in comprehending most of the items of questionnaires. Twenty-one patients were excluded due to previous limb amputation,16 because of missing information on barriers to physical activity, and 109 because of missing information concerning healthcare staff’s attitude towards exercise. Hence, finally 608 patients were available for the analyses (Fig 1).

Demographic data, routine laboratory parameters, comorbidities, data on dialysis treatment and vascular access were extracted from patients’ electronic records. Total comorbidity burden was evaluated by the Charlson index, and the general performance status was summarized by the Karnofski score.

The study was approved by the Ethics Committee of the coordinating center (Comitato Etico per Parma, decision n. 7/13, June 11^th^ 2013), as well as by local Ethics Committees of all participating centers (Comitato Etico Interaziendale di Reggio Emilia, Comitato Etico Interaziendale Bologna-Imola, Comitato Etico per la Provincia di Ferrara, Comitato Etico Area Vasta Romagna, Comitato Etico Area Vasta Toscana Centro, Comitato Etico Area Vasta Toscana Nord-Ovest, Comitato Etico Area Vasta Toscana Sud-Est, Comitato Etico Azienda Policlinico Umberto I, Comitato Etico Campania Nord, Comitato Etico Campania Sud, Comitato Etico Casa Sollievo della Sofferenza San Giovanni Rotondo, Comitato Etico Interregionale di Bari, Comitato Etico Regione Calabria Sezione Sud).

### Assessment of patients’ level of self-reported physical activity and barriers to exercise

Questionnaires concerning levels of mobility/autonomy and physical functioning, self-reported physical activity, and perceived barriers to physical activity were administered to the patients by the nursing staff during a mid-week dialysis session. All members of the nursing staff in participating centers had previously reviewed the different questionnaire items to be able to assist patients during the interviews.

Physical functioning/ autonomy was evaluated by the Katz independence in daily living questionnaire (Activities of Daily Living questionnaire, ADL), a self-reported test on mobility and performance based on 18 items describing different levels of autonomy in physical activity related to participation in life activities [22]. Self-reported physical functioning was also assessed using the Physical Component Summary (PCS) score of the Medical Outcomes Study 12-item short form health survey (SF-12) [23].

Self-reported physical activity was measured with the 94-item Human Activity Profile (HAP) [24], which includes the assessment of activities across a wide range of energy requirements. In HAP, activity items are ranked based on their estimated energy expenditure, from the lowest to the highest. For each item, the respondent indicates whether he or she is still doing, has stopped doing, or never did the activity in question. Answers to HAP items were used to derive the Maximum Activity Score (MAS) and the Adjusted Activity Score (AAS). The MAS is the highest oxygen-demanding activity (highest item number) that the respondent is able to perform. The AAS is obtained by subtracting from the MAS the total number of activities that a patient has stopped doing and have numbers lower than the MAS of that patient. The AAS is regarded as a measure of usual physical activity level [1], with lower values indicating poorer levels of activity.

Patient-related barriers to physical activity were evaluated based on the questionnaire by Delgado and Johansen [10], which includes questions related to different categories of disease-specific and patient-specific barriers to physical activity. Participants were classified as having endorsed a barrier if they reported that they ‘sometimes’, ‘often’ or ‘always’ experienced that barrier; participants who reported that they ‘never’ experienced a barrier were classified as not endorsing that barrier.

### Assessment of staff’s attitude towards physical activity

A specific questionnaire concerning dialysis healthcare staff’s opinions and practices related to exercise counseling [14] was administered to all nephrologists and nurses in each dialysis center.

### Statistical analysis

Stata SE release 15 (2017, StataCorp, College Station, Tx, US) was used for all the analyses.

We examined the cross-sectional association between the degree of physical activity, expressed by HAP-AAS (dependent variable), with the barriers to physical activity in the patient population, and with the attitude of the healthcare dialysis staff towards exercise in patients (independent variables). The models were adjusted for multiple potential confounding patient-related factors, such as age, gender, body mass index, smoking habit, level of education, working status, dialysis vintage, type of vascular access, serum creatinine, serum albumin, hemoglobin level, C-reactive protein and the presence of comorbidities as summarized by the Charlson index.

The HAP-AAS was analyzed as a continuous variate, using multilevel linear models via restricted maximum likelihood (program [ME] mixed). The multilevel models, in which the dialysis center was fitted as a random factor, were used to account for the correlation between data from patients belonging to the same dialysis center. The attitude of the healthcare dialysis staff towards exercise in patients was collected as an indicator variate (0 = non-proactive attitude and 1 = otherwise) for each respondent), each variate reflecting one item from the questionnaire administered to the staff. The average value of each variate within each dialysis unit, which represented the local proportion of the dialysis staff with a proactive attitude, was used for the statistical analyses, and expressed in standard deviation units. Normality assumption of the dependent variate HAP-AAS was checked by inspecting plots of residuals from the fitted models.

We also performed one additional analysis, in which the presence or absence of barriers to physical activity in the patient population was estimated as one single dichotomous variate, with the variate representing the patient endorsing or not endorsing barriers. To this purpose, we used latent class analysis [25,26] (program [SEM] gsem, lclass] to compute the posterior probability from fitted models of belonging to either one of the two classes, and the prevalence of endorsement of each barrier in either class (see also S1 File). Finally, to examine the effect modification exerted by the probability of belonging to the “Endorsing barriers” class on the relationship between the HAP-AAS and the single items describing dialysis-staff’s attitude towards exercise, we fitted interaction terms in the multilevel linear models mentioned above. For this model we used only the items in the staff questionnaire that were independently correlated with the HAP-AAS. A two-tailed P value less than 0.05 was regarded as statistically significant.

## Results

### Patients

The general characteristics of the patients are reported in Table 1. Fifty-six percent were older than 65 years, and 63% were males. Median dialysis vintage was 47.5 months (interquartile range 20.0–95.8 months). Eighty percent were routinely dialyzed through an arteriovenous fistula; mean duration of the dialysis sessions was 3.9 (SD 0.3) hours, and KT/V was 1.4 (SD 0.3). Notwithstanding a moderate comorbidity burden, with a mean Charlson index of 4.0, average performance status (mean Karnofsky score 84.9 [SD 17.0]) and independence (mean ADL score 5.5 [SD 1.2]) in the study population were reasonably good. However, the average level of self-reported physical activity, as expressed by the mean value of the HAP-AAS, was low compared with standard values (71.6 [SD 7.1]) in age-matched healthy subjects [11].

**Table 1:** Characteristics of the patient population.

Variable	
N	608
Age, years (SD)	64.8 (14.5)
Males (%)	62.7
BMI, Kg/m2 (SD)	24.8 (4.8)
Active smoker, %	13.2
Bachelors degree or higher, %	32.4
Working, %	21.6
Dialysis vintage, months (median [1st-3rd quartile])	47.5 [20.0–95.8]
KT/V (SD)	1.4 (0.3)
Dialysis time, hours (SD)	3.9 (0.3)
Vascular access type	
Arteriovenous fistula, %	80.4
Arteriovenous graft, %	4.1
Temporary central venous catheter, %	2.1
Permanent central venous catheter, %	13.4
Diabetes, %	22.0
Coronary artery disease, %	21.9
Peripheral artery disease, %	31.6
COPD, %	13.2
Heart failure, %	11.3
Charlson index (SD)	4.0 (2.0)
Serum creatinine, mg/dL (SD)	9.3 (2.6)
Serum albumin, g/dL (SD)	3.8 (0.5)
Hemoglobin, g/dL (SD)	11.2 (1.3)
C reactive protein, mg/L (median [1^st^-3^rd^ quartile]	2.0 [0.5–6.8]
Karnofski score (SD)	84.9 (17.0)
ADL score (SD)	5.5 (1.2)
SF-12 PCS (SD)	40.0 (10.9)
SF-12 MCS (SD)	41.9 (10.1)
HAP MAS (SD])	64.4 (20.6)
HAP AAS (median [1^st^-3^rd^ quartile])	48.1 (23.1)

ADL, activity of daily living questionnaire; BMI, body mass index; COPD, chronic obstructive pulmonary disease; HAP-AAS, human activity profile-adjusted activity score; HAP-MAS, human activity profile-maximum activity score; SF-12 MCS, 12-item short-form health questionnaire mental component scale; SF-12 PCS, 12-item short-form health questionnaire physical component scale.

Conversion factors for units: serum albumin (g/dL to g/L) x10; C reactive protein (mg/L to μg/mL) x1; serum creatinine (mg/dL to μmol/L) x88.4; hemoglobin (g/dL to g/L) x 10.

The crude and adjusted relationships between the HAP-AAS and relevant demographic, socio-economic and clinical confounders are shown in Table 2. At multivariable analysis age, female sex, smoking, Charlson index and serum C-reactive protein were negatively and independently correlated with the HAP-AAS. Conversely, a higher level of education and higher values of serum creatinine, but not serum albumin or hemoglobin level, were positively and independently correlated with the HAP-AAS.

**Table 2:** Crude and adjusted relationship between self-reported physical activity of hemodialysis patients and relevant demographic, socio-economic and clinical variables.

Variable	Crude HAP-AAS Difference [95% CI]	P	Adjusted * HAP-AAS Difference [95% CI]	P
** **				
Age (x 10 years)	-7.8 [-9.0 to -6.7]	<0.001	-5.2 [-6.8 to -3.6]	<0.001
Female sex	-8.8 [-12.6 to -5.0]	<0.001	-6.9 [-11.7 to -2.2]	0.004
BMI (x Kg/m^2^)	-0.1 [-0.5 to 0.3]	0.5	-0.3 [-0.7 to -0.2]	0.2
Active smoker	3.6 [-2.0 to 9.2]	0.2	-7.4 [-13.2 to -1.6]	0.01
Bachelors degree or higher	14.6 [10.7 to 18.5]	<0.001	7.0 [2.6 to 11.5]	0.002
Not working	-6.6 [-9.0 to -4.2]	<0.001	-2.3 [-5.1 to -0.4]	0.09
Dialysis vintage (x 12 months)	-0.1 [-0.3 to 0.2]	0.5	-0.2 [-0.4 to 0.0]	0.07
KT/V	-0.4 [-8.1 to 7.4]	0.9	1.0 [-7.1 to 9.2]	0.8
Dialysis time (x 1 hour)	5.6 [-1.1 to 12.2]	0.1	-3.8 [-10.8 to 3.3]	0.3
Vascular access type				
Arteriovenous fistula	6.3 [1.4 to 11.3]	0.01	1 (reference)	
Arteriovenous graft	0.1 [-9.7 to 9.9]	0.9	-3.4 [-15.0 to 8.2]	0.6
Temporary central venous catheter	0.9 [-12.2 to 14.0]	0.9	-1.8 [-17.5 to 13.8]	0.8
Permanent central venous catheter	-9.0 [-14.6 to -3.3]	0.002	-5.5 [-11.6 to 0.5]	0.07
Charlson index	-3.5 [-4.5 to -2.5]	<0.001	-1.6 [-2.6 to -0.6]	0.002
Serum creatinine (x mg/dL)	3.4 [2.8 to 4.1]	<0.001	1.8 [0.9 to 2.7]	<0.001
Serum albumin (x g/dL)	9.9 [6.4 to 13.5]	<0.001	2.8 [-2.1 to 7.8]	0.3
Hemoglobin (x g/dL)	1.6 [0.1 to 3.1]	0.04	0.5 [-1.1 to 2.0]	0.6
C-reactive protein (x mg/L)	-0.2 [-0.3 to -0.1]	<0.001	-0.2 [-0.3 to -0.1]	0.007

HAP-AAS, Human Activity Profile–Adjusted Activity Score; BMI, body mass index.

*After including all variables in the model.

### Barriers hindering physical activity

Fatigue (64%) and pain (27%) on dialysis days, lack of time on dialysis days (33%), lack of motivation (41%), feeling to have too many medical problems (29%), sadness (29%), feeling of helplessness (28%), and fear of getting hurt (28%) were the most prevalent barriers among dialysis patients (S1 Table). The same barriers, except lack of time on dialysis days, were negatively and independently correlated with the HAP-AAS at multivariable analysis (Table 3) after adjusting for confounders (adjusted decrease in the HAP-AAS due to the presence of a given barrier ranging between -5.1 [“Feeling too old”, 95% Confidence Interval: -9.4 to -0.8] and -15.6 [“Ulcers on legs and feet”, 95% CI: -24.8 to -6.5]).

**Table 3:** Crude and adjusted relationship between self-reported physical activity of hemodialysis patients and specific barriers to exercise.

	Crude HAP-AAS difference		Adjusted HAP-AAS difference*	
Barriers	Estimate (95% CI)	P	Estimate (95% CI)	P
No place to exercise	-6.3 (-11.7 to -1.0)	0.02	-4.6 (-9.4 to 0.3)	0.06
No safe place to exercise	-3.5 (-8.9 to 1.9)	0.2	-2.3 (-7.1 to 2.4)	0.3
Don’t want to be seen	-3.2 (-12.5 to 6.1)	0.5	1.4 (-6.7 to 9.6)	0.7
No exercise partner	-6.1 (-11.3 to -1.0)	0.02	-2.1 (-6.7 to 2.6)	0.4
Fatigue on dialysis days	-9.2 (-13.2 to -5.3)	<0.001	-6.2 (-9.7 to -2.7)	0.001
Fatigue on non-dialysis days	-13.2 (-17.5 to -9.0)	<0.001	-7.9 (-12.0 to -3.8)	<0.001
Pain on dialysis days	-14.8 (-19.0 to -10.7)	<0.001	-10.1 (-13.9 to -6.4)	<0.001
Pain on non-dialysis days	-17.1 (-21.8 to -12.4)	<0.001	-9.5 (-13.9 to -5.1)	<0.001
Lack of time on dialysis days	4.5 (0.3 to 8.7)	0.03	-1.5 (-5.4 to 2.4)	0.4
Lack of time on non-dialysis days	2.7 (-3.2 to 8.7)	0.4	0.7 (-4.8 to 6.2)	0.8
Too many medical appointments	-3.7 (-9.3 to 2.0)	0.2	-3.9 (-8.9 to 1.2)	0.1
I’m not willing to	-6.5 (-10.4 to -2.7)	0.001	-3.7 (-7.1 to -0.2)	0.04
Feeling too old	-16.4 (-20.9 to -11.8)	<0.001	-5.1 (-9.4 to -0.8)	0.02
Shortness of breath	-12.7 (-17.2 to -8.1)	<0.001	-7.0 (-11.2 to -2.9)	0.001
Fear of getting hurt	-13.4 (-17.6 to -9.2)	<0.001	-6.8 (-10.8 to -2.9)	0.001
Sadness	-8.7 (-12.9 to -4.4)	<0.001	-5.5 (-9.3 to -1.7)	0.005
Feeling of helplessness	-12.0 (-16.2 to -7.8)	<0.001	-7.0 (-10.8 to -3.1)	<0.001
Inability to travel	-23.5 (-28.0 to -19.1)	<0.001	-14.9 (-19.1 to -10.7)	<0.001
Too many medical problems	-14.9 (-19.1 to -10.7)	<0.001	-6.7 (-10.8 to -2.6)	0.001
Chest pain	-16.2 (-23.2 to -9.3)	<0.001	-12.5 (-18.5 to -6.5)	<0.001
Ulcers on legs and feet	-13.0 (-22.6 to -3.3)	0.009	-15.6 (-24.8 to -6.5)	0.001
Family concern	-7.2 (-13.0 to -1.4)	0.02	-6.2 (-11.2 to -1.2)	0.02
Physician concern	-17.4 (-30.9 to -3.8)	0.01	-12.3 (-24.8 to 0.2)	0.05

HAP-AAS, Human Activity Profile–Adjusted Activity Score.

*Adjusted for age, gender, body mass index, smoking status, level of education, working status, dialysis vintage, type of vascular access, Charlson index, serum creatinine, serum albumin, hemoglobin level, C-reactive protein. Coefficients represent the difference in HAP-AAS between patients endorsing and patients not endorsing a given barrier.

### Attitude of the healthcare staff towards physical activity

Mean age of the nephrologists (N = 79) in the dialysis units was 47 (SD 9) years, and mean age of the members of the nursing staff (N = 251) was 46 (SD 8) years.

As shown in S2 Table, members of the healthcare staff showed widespread awareness of the risks of a sedentary lifestyle in the general population (99.4%), and endorsed the potential benefits of increasing the level of physical activity in dialysis patients (96.7%). However, 54.6% of the respondents felt that they lacked time to discuss the issue of physical activity with their patients, and 61.2% felt that participating in exercise had lower priority compared to other medical issues. Moreover, there appeared to be a widespread lack of self-confidence in the ability to provide adequate counseling (50.3% of the respondents), as well as a relatively low interest towards receiving feedback by patients regarding their effective participation in exercise (48.8% of the respondents).

### Impact of healthcare staff’s attitude on the risk of inactivity of dialysis patients

Table 4 shows that after adjusting for confounders, healthcare staff’s perception of lacking time to discuss the issue of exercise, the belief that physical activity is not as important as other medical issues, lack of self-confidence in the ability to provide adequate counseling and failure to ask patients about their participating in exercise were significantly and negatively correlated with patients’ self-reported level of physical activity, as estimated by the HAP-AAS (adjusted change in the HAP-AAS due to the presence of a given staff attitude [per one SD unit increase] ranging between -3.6 [“I don’t trust my capability to discuss the issue of physical exercise with patients”, 95% CI: -6.8 to -0.4] and -5.0 [“I do not have time to discuss the issue of physical exercise with patients on dialysis”, 95% CI: -8.9 to -1.0]).

**Table 4:** Crude and adjusted relationship between self-reported physical activity of hemodialysis patients and specific items describing healthcare personnel’s attitude towards exercise.

	Crude HAP-AAS Difference			Adjusted HAP-AAS Difference*	
Items	Estimate (95% CI)	P		Estimate (95% CI)	P
I do not have time to discuss the issue of physical exercise with patients on dialysis	-4.0 (-8.1 to 0.2)	0.06		-5.0 (-8.9 to -1.0)	0.02
I do not believe that patients on dialysis are interested in the issue of physical exercise	-3.1 (-7.1 to 0.9)	0.1		-2.2 (-5.8 to 1.4)	0.2
I do not believe that physical exercise is important (or is as important as other medical issues)	-3.0 (-6.8 to 0.8)	0.1		-3.7 (-7.2 to -0.1)	0.04
I do not believe that it is physician’s or nurse’s role providing advice on physical exercise to patients on dialysis	-4.0 (-8.5 to 0.6)	0.09		-3.3 (-7.2 to 0.6)	0.09
I do not trust my capability to discuss the issue of physical exercise with patients	-4.3 (-8.1 to -0.5)	0.03		-3.6 (-6.8 to -0.4)	0.03
I do not often ask patients about physical exercise	-3.2 (-7.1 to 0.7)	0.09		-3.6 (-7.1 to -0.1)	0.04

HAP-AAS, Human Activity Profile–Adjusted Activity Score.

* Adjusted for age, gender, body mass index, smoking status, level of education, working status, dialysis vintage, type of vascular access, Charlson index, serum creatinine, serum albumin, hemoglobin level, C-reactive protein. Coefficients represent difference in the HAP-AAS per one SD unit increase of a given item describing staff attitude.

**Fig 2:**
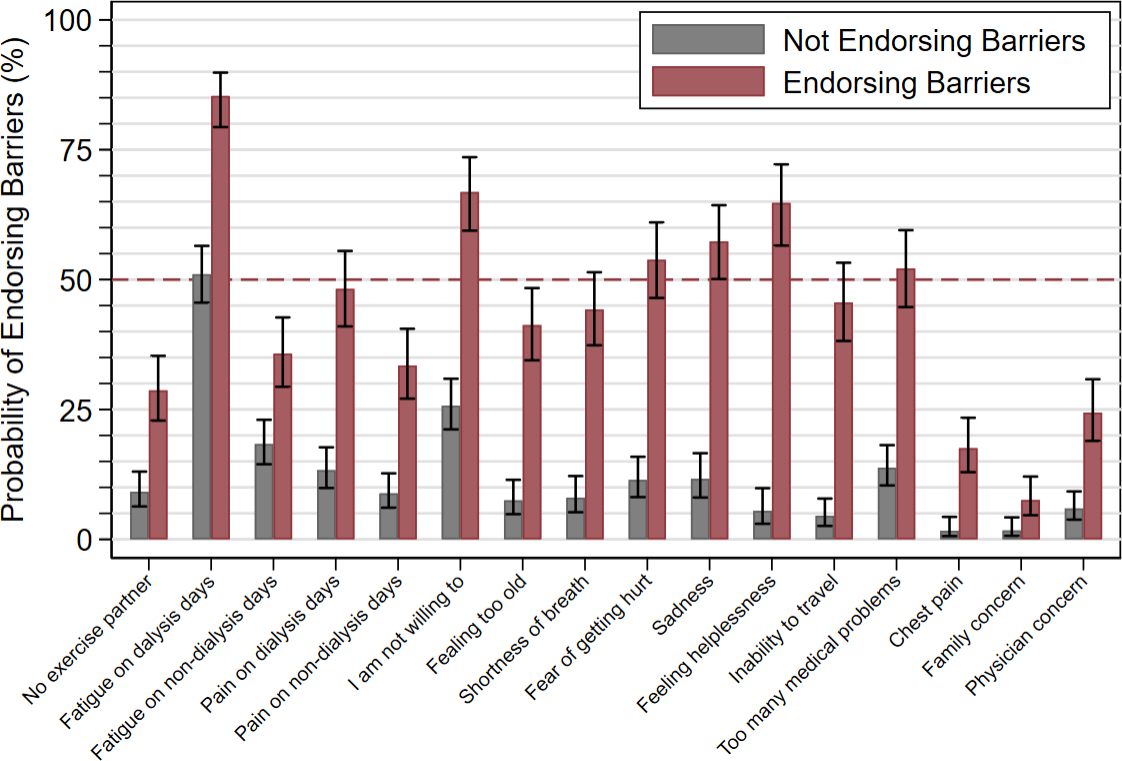
Probability of endorsing barriers to physical activity according to the categorization of hemodialysis patients into “endorsing barriers” and “not endorsing barriers” according to latent class analysis. Bar charts depict predicted values and 95% confidence intervals for the probability of endorsing a given barrier to physical activity, if the patient belongs to the class of “Endorsing barriers” (red bars) or to the class of “Not endorsing barriers” (gray bars). The horizontal dashed line represents 50% probability. It can be seen that the “Endorsing barriers” class was associated with a high (>50%) predicted probability of endorsing a number of barriers. For instance, if the patient is a barrier endorser, then the probability of endorsing "Sadness", and "Feeling helpless" is significantly greater than 50% (i.e., the 95% confidence intervals do not cross the dashed line); if the patient is not a barrier endorser, then the probability of endorsing "Sadness", and "Feeling helpless" is low.

However, the impact of healthcare staff’s attitude on the risk of inactivity of dialysis patients varied according to whether or not the patients endorsed barriers. We examined this phenomenon by categorizing patients into two classes by latent class analysis. The estimated probability of being in the “Endorsing barriers” class in the whole patient population was 38.2% [95% CI: 32.6 to 44.1%]. Fig 2 reports how the predicted probabilities of endorsing a given barrier differed according to the patients’ belonging to either class. It can be seen that if a patient belongs to the “Endorsing barriers” class, there is a high (>50%) probability of endorsing a number of barriers. For instance, if the patient is a barrier endorser, then the probability of endorsing "Sadness", and "Feeling helpless" is significantly greater than 50% (i.e., the 95% CIs do not cross the dashed line); if the patient is not a barrier endorser, then the probability of endorsing "Sadness", and "Feeling helpless" is low.

**Fig 3:**
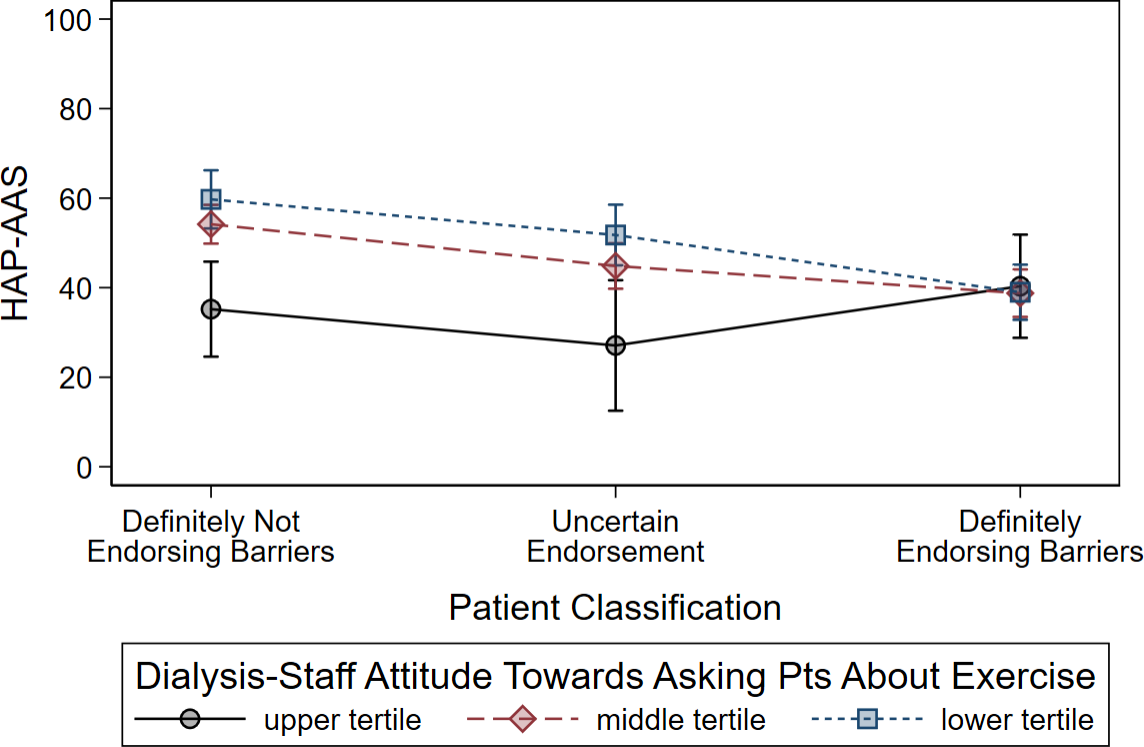
Illustrative example of the interaction between the probability of patients endorsing barriers to exercise (based on latent class analysis) and the healthcare staff’s level of interest towards receiving feedback from patients on physical activity. Dots represent fitted means from the multiple regression models (see text), vertical bars represent 95% confidence intervals. Dots are connected by lines for the purpose of helping visual comparisons between means. Patients were categorized into three classes according to different values (i.e., <5%, 5–95%, >95%) of the posterior probability of being barrier endorsers according to latent class analysis. Healthcare staff’s level of interest towards receiving feedback from patients was categorized according to tertiles of the distribution of dialysis center-averaged values. Upper tertile indicates highest interest by the dialysis staff towards patients’ physical exercise; Lower tertile indicates lowest interest. Unlike in patients who were definitely barrier endorsers, in other patient categories dialysis staff attitude did make a difference, as the dialysis staffs with the highest interest in physical exercise (blue line) was associated with an increase in thethe HAP-AAS by nearly 30 points compared to the dialysis staff with the lowest interest in physical exercise (black line).

We found a statistically significant effect modification of the probability of belonging to the “Endorsing barriers” class on the relationship between the dialysis staff’ attitude towards exercise and the HAP-AAS. This effect modification was examined by interaction coefficients, which showed that a definite beneficial effect of a proactive dialysis staff attitude was evident only in the patients belonging to the “Not endorsing barriers” class: adjusted interaction of barriers with “I do not have time to discuss the issue of physical exercise with patients on dialysis”, P = 0.023; with “I do not believe that exercise is important (or is as important as other medical issues)”, P = 0.012; with “I do not believe that it is physician’s or nurse’s role providing advice on exercise to patients on dialysis”, P = 0.030; with “I do not often ask patients about exercise”, P = 0.001. In order to provide a visual appraisal of the interaction coefficients, we divided the patients into three groups according to the probability of belonging to “Endorsing barriers” class being <5% (288 patients [47%]), 5–95% (158 patients [26%]), and >95% (162 patients [27%]), and performed the analysis whose results are plotted in Fig 3 (this illustrative example concerns the most significant interaction coefficient, namely with “I do not often ask patients about exercise”). Unlike in patients who were definitely barrier endorsers, in other patient categories dialysis staff’s attitude did make a difference, as the dialysis staff with the highest interest in exercise was associated with a nearly 30-point increase in the HAP-AAS compared to the dialysis staff with the lowest interest in exercise.

## Discussion

The main finding of this study is that, in a representative sample of Italian dialysis centers, several barriers to exercise and a non-proactive attitude by the healthcare staff impact significantly and negatively on patients’ self-reported levels of physical activity, while a definite beneficial effect of a proactive staff’s attitude is evident only in patients not endorsing barriers.

Previous work available on this issue is limited, and mainly based on qualitative assessments [13,15,19,27,28]. Jhamb and co-workers [13] emphasized that a supportive staff behavior was a powerful motivator to performing exercise in dialysis patients; intriguingly, these investigators also suggested a positive interaction between a proactive attitude of healthcare providers and the clinical setting (e.g., availability of exercise programmes, trained supervisors) in stimulating patients’ willingness to increase physical activity. Conversely, exercise counseling is low among both nephrologists and nurses [14,17,27]. Indeed, Painter et al [27] reported that 24% of their surveyed dialysis staff never or seldom encouraged patients to participate in physical activity, and 34% never asked patients about physical barriers to exercise. An overaccomodating staff’s behavior, lack of self-confidence in counseling ability and lack of facilities were also shown by the same group [28] as important factors increasing the risk of patient inactivity. In a study by Delgado and Johansen [17], the belief that exercise is not as important as other medical issues, that most patients would not increase their level of physical activity despite being counseled to do so, and lack of self-confidence in the ability to discuss the issue of physical activity were independent predictors of low counseling probability among nephrologists. We also found that nearly 60% of the members of the healthcare staff in dialysis units thought that patients would probably not increase their levels of exercise even if advised to do so; moreover, more than 60% believed that promoting exercise in patients had lower priority compared to other medical issues. Furthermore, 63% believed that providing advice on exercise is not part of physicians’ or nurses’ role, and self-confidence in the ability to counsel patients was generally low (50%). The perception that counseling on exercise would impose excessive workload on the healthcare staff was also common (55% of the respondents). The combination of these beliefs conceivably explains [14,19] the relatively passive attitude of the healthcare providers in dialysis units towards asking patients about their levels of physical activity (asking prevalence 51%). Importantly, we found that a non-proactive staff’s attitude was negatively correlated with the level of self-reported physical activity.

Among patient-perceived barriers, fatigue and pain on dialysis days, lack of motivation, feeling helpless, and having too many medical problems emerged as major obstacles to physical exercise encountered by hemodialysis patients in this and previous studies [9,10,12,13,19,27]. Thus, contrasting these barriers through an encouraging and supporting attitude by the healthcare staff may represent a potentially cost-effective strategy to increase patients’ level of physical activity and quality of life [3,15] and to decrease their cardiovascular risk [4,5]. In fact, evidence from the Dialysis Outcomes and Practice Patterns Study [29] shows that the level of aerobic activity is positively associated with health-related quality of life in dialysis patients, while it is inversely associated with depression and overall mortality. Moreover, other studies [30–34] demonstrated that the implementation of structured exercise programmes in dialysis facilities increases patients’ quality of life and decreases depression. Furthermore, the recently published multicenter randomized Exercise Introduction to Enhance Performance in Dialysis trial [21] showed very convincingly that a personalized 6-month walking programme at home increased significantly the cognitive function and the social interaction scores in hemodialysis patients. Thus, support and empowerment in dialysis units could increase patient participation in exercise, which could in turn increase their perception of well-being and favor persistence in an active lifestyle. In this respect, the implementation of intradialytic exercise has been shown to be associated with significant improvements in patients’ physical functioning and well-being [35], and there is established consensus that intradialytic exercise programmes are sustainable [36]. Indeed, the importance of a direct involvement of specialized professionals (e.g., physiotherapists and exercise physiologists) to supervise and assist patients during intradialytic exercise has been underscored [18,36]. As general conditions and physical functioning may be highly variable among hemodialysis patients, it is crucial that exercise programmes be individualized [20,31]. In fact, tailoring exercise programmes to patients’individual tolerance may help decrease the impact of pain, fatigue, or depression [15].

Exploiting the first two dialysis hours during each session to deliver supervised aereobic or resistance training, or a combination of the two, is also a convenient way to increase adherence to exercise in patients [35]. Moreover, dialysis hours represent a privileged time period for informing patients concerning the benefits of increasing their level of physical activity, and to decrease patients’ boredom during the dialysis session [13]. During dialysis hours, an encouraging attitude by dialysis staff members directed to patients’ empowerment appears as one of the most important issues in promoting an active behavior [13]. However, a proactive staff’s attitude may be frustrated by the negative impact of physical and psychological barriers hindering patients’ adoption of an active behavior. In this respect, a study employing the Theoretical Domain Framework to focus on barriers and facilitators of intradialytic exercise [15] revealed that dialysis staff members expressed a strong need for a specific training to increase their knowledge and therefore be able to overcome patient-related barriers; specularly, patients perceived the lack of responsibility for the intradialytic exercise programme among staff members as a powerful barrier. Another study [19] evaluated the impact of a 3-month structured programme of patient and nursing staff education, equipment modification and introduction of patient motivation schemes on patients’ uptake of intradialytic exercise. Although there was a significant increase in the participation in intradialytic exercise, there was also an increase in the proportion of patients feeling that exercise is not suitable for people with multiple health problems. Thus, patients in particularly poor health condition and endorsing multiple barriers to exercise may benefit less from a proactive staff’s attitude than patients in better general conditions and endorsing less barriers. This was in fact an important finding of our study, where a beneficial effect of a proactive staff’s attitude was evident only in those patients who were not definitely barrier endorsers.

We acknowledge that our study has limitations. Firstly, being observational and cross-sectional in nature, it can only generate hypotheses concerning the causative role of specific staff’s attitudes towards exercise on the implementation of a more or less active lifestyle by hemodialysis patients. Secondly, we did not use objective measures of physical activity in our patient population; in fact, indexes of self-reported physical activity may be biased towards lower estimates given the frequent occurrence of depression and lack of motivation in hemodialysis patients. However, HAP was very well correlated with the level of 7-day physical activity as measured with an accelerometer [22], and was used successfully to quantify the level of physical activity in a large cohort of incident hemodialysis patients [1]. Thirdly, information on dialysis staff’s attitude from some dialysis centers was missing. However, we had an overall 85% response rate, that is above the 80% threshold value regarded as a high response rate in surveys.

### Conclusions

Notwithstanding these limitations, the large cohort of patients and the wide distribution of dialysis units across Italian peninsular areas suggest that the results of this study may also apply to the population of hemodialysis patients in other Western countries. Investing resources in staff training on exercise counseling in dialysis units may represent a potentially cost-effective strategy to decrease sedentarism in hemodialysis patients, also ameliorating health outcomes and quality of life [37] and decreasing morbidity-related costs in this patient population. However, based on our findings, only a fraction of patients, namely those not heavily endorsing physical and psychological barriers, would definitely benefit from this intervention; in fact, in our study, nearly 62% of the patient population were not definite barrier endorsers. On the other hand, in patients heavily endorsing barriers, a 'stepped care' approach could be envisaged, whereby staff’s efforts should be directed firstly to overcome psychological (i.e., sadness, sense of helplessness or lack of motivation) barriers; subsequently specific low-intensity exercise programmes could be tailored for patients with multiple physical barriers. The perception of lacking dedicated time may be addressed by bulding specialized teams, including only few motivated staff members and physiotherapists within each dialysis unit, to deliver counseling activity.

Further prospective studies addressing the impact of implementing programmes of dialysis staff training on patients’ physical activity levels and related changes in morbidity are awaited to test the hypotheses generated by this study.

## Supporting information

S1 File Latent class model. Methodological explanation.
(DOCX)

S2 File Study group physical exercise in CKD. List of names and e-mail addresses of all members of the Study Group Physical Exercise in CKD of the Italian Society of Nephrology.
(XLSX)

S1 Table Prevalence of different barriers to physical activity in the patient population. (DOCX)

S2 Table Attitude of the healthcare dialysis staff towards physical exercise in patients. (DOCX)

S1 Dataset Data set. Full data set of experimental data used for all analyses.
(XLS)
