# Correction: Interaction of healthcare staff’s attitude with barriers to physical activity in hemodialysis patients: A quantitative assessment

**DOI:** 10.1371/journal.pone.0198987

**Published:** 2018-06-20

**Authors:** Giuseppe Regolisti, Umberto Maggiore, Alice Sabatino, Ilaria Gandolfini, Sarah Pioli, Claudia Torino, Filippo Aucella, Adamasco Cupisti, Valentina Pistolesi, Alessandro Capitanini, Giorgia Caloro, Mariacristina Gregorini, Yuri Battaglia, Marcora Mandreoli, Lucia Dani, Giovanni Mosconi, Vincenzo Bellizzi, Biagio Raffaele Di Iorio, Paolo Conti, Enrico Fiaccadori, on behalf of the Gruppo di Studio “Esercizio fisico nel paziente con insufficienza renale cronica” of the Società Italiana di Nefrologia

Fig 3 and the caption for Fig 3 are incorrect. The authors have provided a corrected version here.

**Fig 3:**
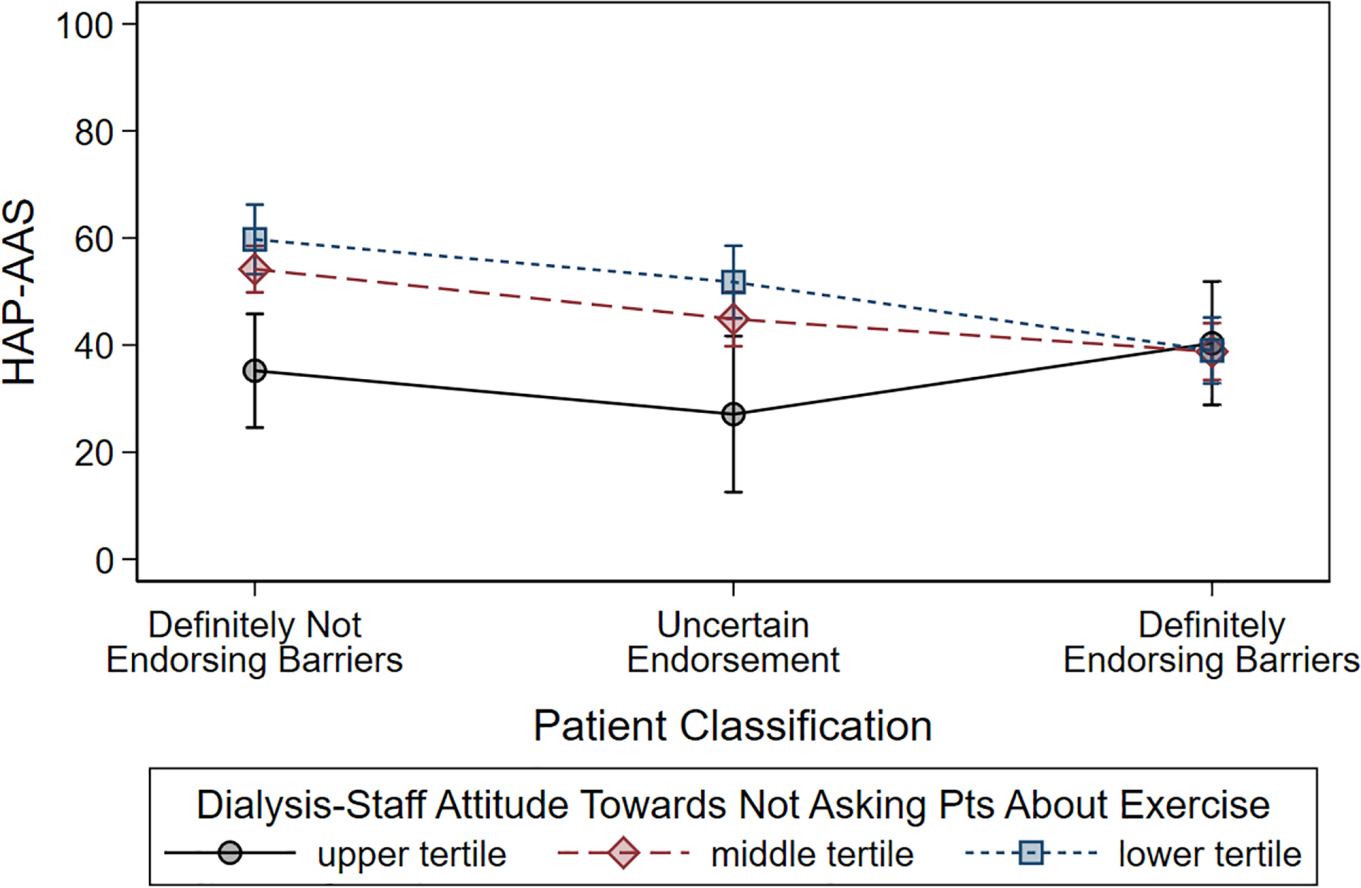
Illustrative example of the interaction between the probability of patients endorsing barriers to exercise (based on latent class analysis) and the healthcare staff’s level of interest towards receiving feedback from patients on physical activity. Dots represent fitted means from the multiple regression models (see text), vertical bars represent 95% confidence intervals. Dots are connected by lines for the purpose of helping visual comparisons between means. Patients were categorized into three classes according to different values (i.e., <5%, 5±95%, >95%) of the posterior probability of being barrier endorsers according to latent class analysis. Healthcare staff’s level of interest towards receiving feedback from patients was categorized according to tertiles of the distribution of dialysis center-averaged values. Upper tertile indicates lowest interest by the dialysis staff towards patients’ physical exercise; lower tertile indicates highest interest. Unlike in patients who were definitely barrier endorsers, in other patient categories dialysis staff attitude did make a difference, as the dialysis staffs with the highest interest in physical exercise (blue line) was associated with an increase in the HAP-AAS by nearly 30 points compared to the dialysis staff with the lowest interest in physical exercise (black line).
