# Supplementary material for: Interaction of healthcare staff’s attitude with barriers to physical activity in hemodialysis patients: A quantitative assessment
Source: PLoS One. 2018 Apr 27;13(4):e0196313. doi: 10.1371/journal.pone.0196313 (PMC5922547; doi:10.1371/journal.pone.0196313)
Supplement: S1 File — Methodological explanation. (DOCX) [file pone.0196313.s001.docx]

**Latent Class Model**

Latent class model can be used to study a heterogeneous population, that is a population consisting of several unidentified groups that behave differently regarding the problem at hand, such as endorsing barriers to exercise. Latent class analysis can make the existence of these groups, namely “Endorsing barriers” and Not endorsing barriers”, apparent. The latent class model is a model for measuring those latent (unobserved) categories by means of a set of observed categorical variables (i.e., the patient's answers to the different items of the questionnaire on barriers). These observed variables are considered to be indicators of the underlying concept (the dialysis population consists of a heterogeneous population concerning barrier endorsement).
