# Supplementary material for: Interaction of healthcare staff’s attitude with barriers to physical activity in hemodialysis patients: A quantitative assessment
Source: PLoS One. 2018 Apr 27;13(4):e0196313. doi: 10.1371/journal.pone.0196313 (PMC5922547; doi:10.1371/journal.pone.0196313)
Supplement: S1 Table — (DOCX) [file pone.0196313.s003.docx]

S1 Table. Prevalence of different barriers to physical activity in the patient population

| **Barriers** |  | **%**  **N = 608** |
| --- | --- | --- |
|  |  |  |
| No place to exercise |  | 15.6 |
| No safe place to exercise |  | 15.8 |
| Don’t want to be seen |  | 4.4 |
| No exercise partner |  | 16.6 |
| Fatigue on dialysis days |  | 64.1 |
| Fatigue on non-dialysis days |  | 25.0 |
| Pain on dialysis days |  | 26.6 |
| Pain on non-dialysis days |  | 18.3 |
| Lack of time on dialysis days |  | 33.2 |
| Lack of time on non-dialysis days |  | 12.2 |
| Too many medical appointments |  | 13.3 |
| I’m not willing to |  | 41.4 |
| Feeling too old |  | 20.4 |
| Shortness of breath |  | 21.9 |
| Fear of getting hurt |  | 27.6 |
| Sadness |  | 29.1 |
| Feeling of helplessness |  | 28.1 |
| Inability to travel |  | 20.2 |
| Too many medical problems |  | 28.5 |
| Chest pain |  | 7.7 |
| Ulcers on legs and feet |  | 3.9 |
| Family concern |  | 13.0 |
| Physician concern |  | 2.3 |
