# Supplementary material for: Interaction of healthcare staff’s attitude with barriers to physical activity in hemodialysis patients: A quantitative assessment
Source: PLoS One. 2018 Apr 27;13(4):e0196313. doi: 10.1371/journal.pone.0196313 (PMC5922547; doi:10.1371/journal.pone.0196313)
Supplement: S2 Table — (DOCX) [file pone.0196313.s004.docx]

Table S2. Attitude of the healthcare dialysis staff towards physical exercise in patients

| **Item** |  | **%**  **N=330** |
| --- | --- | --- |
|  |  |  |
| 1. Sedentary lifestyle is an important health risk factor for the general population |  | 99.4 |
| 2. Increasing physical exercise is beneficial for most people |  | 97.9 |
| 3. Physical exercise is useful for patients on dialysis |  | 96.7 |
| 4. I am concerned about the risks of exercise in patients on dialysis |  | 18.2 |
| 5. I do not believe that most of the patients on dialysis would increase physical exercise even if advised to do so |  | 59.4 |
| 6. I do not have time to discuss the issue of physical exercise with patients on dialysis |  | 54.6 |
| 7. I do not believe that patients on dialysis are interested in the issue of physical exercise |  | 37.0 |
| 8. I do not believe that physical exercise is important (or is as important as other medical issues) |  | 61.2 |
| 9. I do not believe that it is physician’s or nurse’s role providing advice on physical exercise to patients on dialysis |  | 63.3 |
| 10. I do not trust my ability to discuss the issue of physical exercise with patients |  | 50.3 |
| 11. I do not often ask patients about physical exercise |  | 48.8 |
| 12. I do not often counsel my patients about physical exercise |  | 66.1 |
| 13. I do not often ask and counsel about physical exercise |  | 50.3 |
